# Impact of Glucosamine Supplementation on Gut Health

**DOI:** 10.3390/nu13072180

**Published:** 2021-06-24

**Authors:** Jessica M. Moon, Peter Finnegan, Richard A. Stecker, Hanna Lee, Kayla M. Ratliff, Ralf Jäger, Martin Purpura, Carolyn M. Slupsky, Maria L. Marco, Craig J. Wissent, Jason Theodosakis, Chad M. Kerksick

**Affiliations:** 1 Exercise and Performance Nutrition Laboratory, School of Health Sciences, Lindenwood University, St. Charles, MO 63301, USA; jmm805@lindenwood.edu (J.M.M.); rstecker@lindenwood.edu (R.A.S.); kratliff@lindenwood.edu (K.M.R.); 2 Department of Food Science & Technology, University of California, Davis, CA 95616, USA; pfinnegan@ucdavis.edu (P.F.); hnlee@ucdavis.edu (H.L.); cslupsky@ucdavis.edu (C.M.S.); mmarco@ucdavis.edu (M.L.M.); 3 Increnovo, LLC, Milwaukee, WI 53202, USA; ralf.jaeger@increnovo.com; 4 Department of Nutrition, University of California, Davis, CA 95616, USA; martin.purpura@increnovo.com; 5 Jamieson Wellness Inc., Windsor, ON N8N 5E7, Canada; cwissent@jamiesonlabs.com; 6 Dr. Theo’s, Inc., Tucson, AZ 85750, USA; drjtheo@aol.com

**Keywords:** microbiota, gut health, diversity, metabolomics, stool, gastrointestinal

## Abstract

Glucosamine (GLU) is a natural compound found in cartilage, and supplementation with glucosamine has been shown to improve joint heath and has been linked to reduced mortality rates. GLU is poorly absorbed and may exhibit functional properties in the gut. The purpose of this study was to examine the impact of glucosamine on gastrointestinal function as well as changes in fecal microbiota and metabolome. Healthy males (*n* = 6) and females (*n* = 5) (33.4 ± 7.7 years, 174.1 ± 12.0 cm, 76.5 ± 12.9 kg, 25.2 ± 3.1 kg/m^2^, *n* = 11) completed two supplementation protocols that each spanned three weeks separated by a washout period that lasted two weeks. In a randomized, double-blind, placebo-controlled, crossover fashion, participants ingested a daily dose of GLU hydrochloride (3000 mg GlucosaGreen^®^, TSI Group Ltd., Missoula, MT, USA) or maltodextrin placebo. Study participants completed bowel habit and gastrointestinal symptoms questionnaires in addition to providing a stool sample that was analyzed for fecal microbiota and metabolome at baseline and after the completion of each supplementation period. GLU significantly reduced stomach bloating and showed a trend towards reducing constipation and hard stools. Phylogenetic diversity (Faith’s PD) and proportions of *Pseudomonadaceae*, *Peptococcaceae*, and *Bacillaceae* were significantly reduced following GLU consumption. GLU supplementation significantly reduced individual, total branched-chain, and total amino acid excretion, with no glucosamine being detected in any of the fecal samples. GLU had no effect on fecal short-chain fatty acids levels. GLU supplementation provided functional gut health benefits and induced fecal microbiota and metabolome changes.

## 1. Introduction

Glucosamine is an amino sugar found naturally in the cartilage and fluid around the joints and is also contained in crustacean exoskeletons and some fungi. Glucosamine is one of the most frequently supplemented ingredients consumed by U.S. adults [1], particularly due to its potential anti-inflammatory and anti-apoptotic effects on articular cartilage and bone [2]. In clinical trials, glucosamine has frequently shown beneficial outcomes in joint pain, joint function, and other related clinical outcomes [3,4,5,6,7]. Additionally, epidemiology data has pointed to lower mortality rates in individuals who were supplementing with glucosamine [8,9], a surprising outcome when considering its most common application (pain relief in osteoarthritic individuals) is in a population that typically presents with multiple comorbidities [1,6,10]. A cohort study by Pocobelli [9] of 77,719 people who averaged 10 years of glucosamine use were found to have five-year mortality rates that were significantly lower than non-glucosamine users. In addition, Ma et al. [10] followed 466,093 participants without cardiovascular disease (CVD) at baseline in the UK biobank for a median of seven years. Glucosamine use was associated with significantly lower risks of CVD events and death. Moreover, other data involving glucosamine has led people to question a greater role for glucosamine in human health. For example, large prospective cohort studies [11,12,13,14] involving glucosamine alone and in combination with chondroitin have suggested a reduced association of risk for developing colorectal cancers. Additionally, animal [15] and human [16] models have reported improvements in inflammatory bowel disease after glucosamine administration. In considering this data, other potential roles of glucosamine in human health and associated mechanisms of action may be evident, and other mechanisms of action, besides the proposed biochemical mechanisms related to the joints (i.e., cytokine and enzyme changes in the joints’ chondrocytes, synoviocytes and synovial fluid), should be investigated to elucidate the potential benefits seen systemically by glucosamine supplementation.

Within the human digestive tract, only 10–12% of the ingested glucosamine is absorbed [17,18,19]. In fact, glucosamine absorption is so low it has previously been used as a coating agent for various drugs [20,21]. Due to its simplicity as an amino sugar, more than 50% of ingested glucosamine is metabolized by members of the intestinal microbiota before it is absorbed into the bloodstream [19]. For these reasons, evidence has started to grow indicating that glycosaminoglycans such as glucosamine and chondroitin may have potential gut health benefits. In consideration of a potential mechanism, multiple authors have reported that glucosamine (and chondroitin) may function in various roles in the production of anti-inflammatory compounds that are known to be increased in various inflammatory and metabolic diseases [21,22,23,24].

Evidence that has examined the potential ability of glucosamine availability to change the abundances of various strains and species of bacteria in the microbiome is limited. Shmagel et al. [21] recently published a systematic review of evidence from animal and human studies on the effects of glucosamine and chondroitin sulfate on gut microbial composition. Of the included studies in this review, only one study, which lacked a placebo group, examined the impact of glucosamine on the gut microbiome. In this study, Coulson et al. [25] randomized 38 subjects with knee osteoarthritis to consume orally on a daily basis 3000 mg of glucosamine sulfate or 3000 mg of green lipped muscle extract (*Perna canaliculus)* for 12 weeks. Fecal microbial analysis before and at the end of the intervention showed no changes in total microbial diversity, the absolute abundance of *Staphylococcus*, *Bacterioides*, and *Clostridium* genera tended to decrease while *Lactobacillus*, *Streptococcus*, *Coliforms*, and *Eubacterium* genera tended to increase after supplementation. In conjunction with these changes, Coulson et al. [25] also reported a significant improvement in osteoarthritis symptom measurements and gastrointestinal symptoms. However, greater conclusions from this study are underpinned by its lack of a placebo or appropriate control group. Most recently, Navarro et al. [26] observed that oral co-administration of glucosamine hydrochloride (derived from crab and shrimp) and chondroitin sulfate significantly modulated nine different genera after a 14-day supplementation using a placebo-controlled, crossover design. Specifically, abundances of *Lachnospiraceae*, *Prevotellaceae*, and *Desulfovibrio* were increased while *Bifidobacterium* and *Christensenellaceae* were decreased. To date, no clinical study has investigated the ability of glucosamine by itself to impact changes in stool, gastrointestinal symptoms and in the quantity and function of bacteria in the microbiome in comparison to a control group. For these reasons, the purpose of this study was to examine the impact of three weeks of vegetarian, non-shellfish-derived glucosamine hydrochloride supplementation on changes in the fecal microbiome, stool function, and gastrointestinal symptoms. It was hypothesized that glucosamine supplementation would yield significant improvements in gastrointestinal function and be responsible for significant changes in the genera of bacteria found in the fecal microbiome.

## 2. Materials and Methods

### 2.1. Experimental Design

Healthy males and females participated in this randomized, double-blind, placebo-controlled, cross-over study design. As this was a pilot study designed to identify the potential for a 3-week regimen of daily non-shellfish (fermented) glucosamine supplementation, no formal sample size analysis was completed prior to the investigation. Participants were instructed to report to the laboratory after observing an overnight (8 to 10-h) fast while maintaining adequate hydration. Participants were asked to abstain from exercise and avoid alcohol and caffeine consumption for 12 h prior to each study visit. All participants first completed an initial eligibility visit during which a comprehensive medical history, consent document, and body mass index (BMI) assessment were completed. Results from the BMI assessment was used to finalize eligibility. Once deemed eligible, participants were provided with supplies to begin the study protocol.

**Figure 1:**
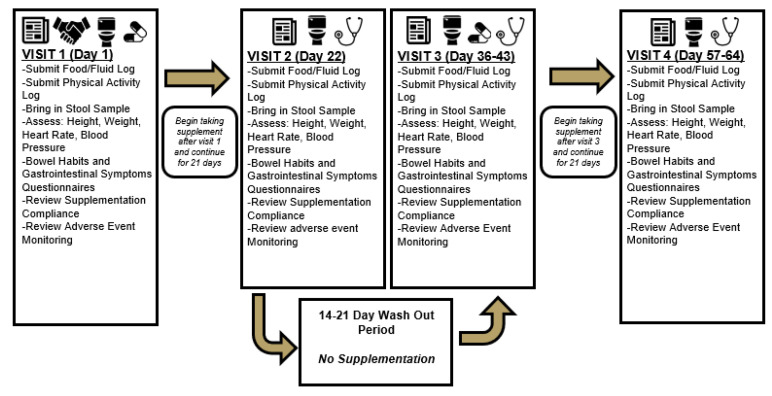
Flow chart of experimental design.

The remainder of the study consisted of two identical patterns of testing with the exception of which supplementation protocol was followed. (See Figure 1). Prior to each visit, food and physical activity logs were completed, encompassing four days prior to the scheduled laboratory visit consisting of two weekend days and two weekdays. Each participant reported to the laboratory for a baseline measurement of height, weight, heart rate, and blood pressure. During this visit, participants completed the Bristol Stool Chart and Gastrointestinal Symptom Rating Scale (GSRS) to assess bowel habits and potential gastrointestinal symptoms. Finally, participants were given instructions on how to collect a stool sample and were instructed to do so within 24 h of each planned study visit. At the completion of this study visit, participants were provided with their first assigned supplement and instructed to begin supplementation 21 days prior to their second visit. Participants were instructed to collect their second stool sample on the day of their final supplementation dose. On the morning of their second study visit, participants reported to the laboratory after following identical pre-study instructions as on previous visits (fasting, abstention from caffeine and exercise). Study participants again completed assessments for height, weight, heart rate, blood pressure, and questionnaires regarding their bowel habits and gastrointestinal symptoms while also providing their second stool sample and turning in completed food and activity logs. A washout period lasting a minimum of 14 days and a maximum of 21 days separated each phase of the crossover. Burns et al. [27] previously reported no difference in fecal DNA content after employing a 14-day washout period between three periods of three weeks of supplementation with a resistant maltodextrin starch.

### 2.2. Study Participants

**Figure 2:**
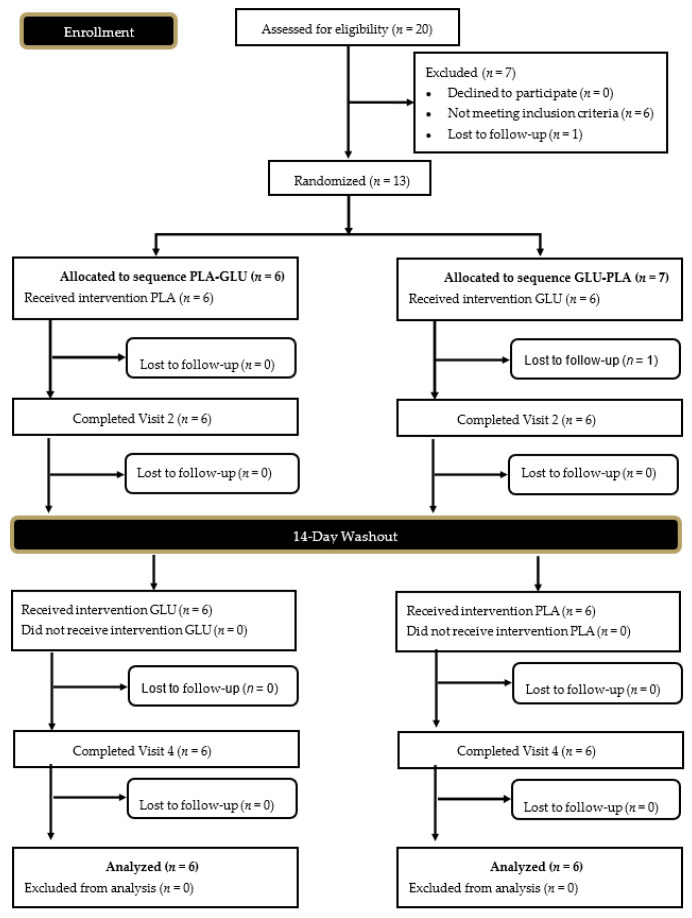
Consolidated Standards of Reporting Trials (CONSORT) Diagram Procedures. Glucosamine (GLU)**,** Placebo (PLA).

A CONSORT diagram for all study recruitment randomization, and project completion is provided as Figure 2. Data collection commenced in January 2019 and was completed in June 2019. A total of six male (age: 32.0 ± 9.6 years, height: 180.5 ± 10.0 cm, weight: 81.0 ± 11.3 kg, body mass index: 24.8 ± 2.8 kg/m^2^) and five female (age: 35.0 ± 5.4 years, height: 166.5 ± 10.1 cm, weight: 71.1 ± 13.8 kg, body mass index: 25.6 ± 3.8 kg/m^2^) participants completed the study protocol. Prior to participation, participants provided their signed informed consent using an IRB approved consent document (Protocol # IRB-19-8, Approval Date 18 October 2018) and registered with the ISRCTN registry (ISRCTN28637234). Participants were randomized into the study protocol if they were between the ages of 18–50 years, had a body mass index between 18.5–27 kg/m^2^, weight stable for the past three months (less than 5% variation in body mass), and were free from all cardiovascular, pulmonary, autoimmune, musculoskeletal, gastrointestinal, psychological, or other diseases or disorders, as reported in their medical history. Participants could not have been currently taking any antibiotics, probiotics, or have indicated that they were actively trying to lose weight which included participating in diets that would impact study outcomes (ketogenic or low carbohydrate diet). Alternatively, participants were excluded if they were: diagnosed with or were being treated for celiac disease, lactose intolerance, digestive insufficiencies, or other gastrointestinal complications such as irritable bowel syndrome, ulcerative colitis, etc.; were a current smoker or had quit within the past six months; were currently using anabolic steroids or any illicit or recreational drugs; or were currently taking any antibiotic or medication known to impact study outcomes. Participants who reported consuming a probiotic or other form of any dietary supplement purported to impact digestion or gut health were required to stop taking that supplement for 30 days before beginning the study trial.

### 2.3. Anthropometrics

Height and weight were measured for every participant without shoes and in minimal clothing using a standardized wall-mounted stadiometer (Tanita, model # HR-200, Tokyo, Japan) and a self-calibrating digital scale (Tanita, model # BWB-627A Class III, Tokyo, Japan). Prior to heart rate and blood pressure measurements, participants rested in a seated position for five minutes. Heart rate was measured on the right arm at the radial artery, placing the index and middle fingers two centimeters proximal to the styloid process of the radius, by a trained research assistant. The number of heart beats was counted over a 30-s period and multiplied by two. Blood pressure was measured on the right arm using a standard blood pressure cuff and sphygmomanometer at the brachial artery. Two measurements were taken and averaged. The right arm was positioned at the level of the heart with the distal edge of the cuff placed two centimeters above the bend in the elbow prior to the cuff being inflated to a value that was 30 mm of mercury (mmHg) higher than the participant’s self-reported blood pressure. The cuff was deflated at approximately 3 mmHg per second until the first and fourth Korotkoff sounds were detected. Both values associated with these events were recorded and used for the blood pressure assessment.

### 2.4. Dietary and Physical Activity Logs

Study participants completed assessments of food intake and physical activity at the beginning and end of each phase of testing. Intake was reported over a four-day period prior to in-lab assessments encompassing two weekdays and two weekend days. All activities of daily living as well as exercise and sleep were self-reported by participants in a 24-h fitness log. All food intake encompassing food type, preparation style, and amount were self-reported on paper by participants for the four-day assessment period.

### 2.5. Supplementation Protocol

In a double-blind manner, a glucosamine supplement or a maltodextrin placebo was distributed in identical opaque capsules to all participants. On a daily basis, participants were instructed to ingest three capsules, each containing 1000 mg of Glucosamine Hydrochloride (GlucosaGreen^®^ TSI Group Ltd. Missoula, MT, USA) or a placebo (maltodextrin) identical in appearance for a total of 21 days. Each visit at the beginning of the supplementation regimen (Visits 1 and 4, respectively; See Figure 1) occurred on or before the first day of supplementation while the final day of supplementation occurred the day before each participant’s follow-up visit for that study period (Visits 2 and 4, respectively). Participants were instructed to ingest the supplement 15–30 min before breakfast with eight fluid ounces of cold water. In the case of a missed dose, participants were instructed to consume the missed dose as soon as possible with a meal. A maltodextrin placebo was used due to previous research that demonstrated a lack of changes in fecal microbiome content after 7.5 [27] and 15 [28] grams of resistant maltodextrin were ingested. The dose of maltodextrin used in the present study was two to five-fold lower, respectively, than these investigations.

### 2.6. Stool Quality and Gastrointestinal Questionnaires

Participants completed electronic (Qualtrics, Inc., Washington, DC, USA) versions of the Bristol Stool Chart [29] and the Gastrointestinal Symptoms Rating Scale (GSRS) [30] at four time points throughout the entire study protocol. The first two questionnaires were completed before and after the first supplementation period while the final two questionnaires were collected before and after the second supplementation period.

Briefly, the Bristol Stool Chart requires participants to classify their bowel movements into one of seven different types of stool. Type 1 was classified as being “Separate hard lumps, like nuts (hard to pass)”, Type 2 being “Sausage-shaped, lumpy, uncomfortable to pass”, Type 3 being “Like a sausage, with cracks on its surface”, Type 4 being “Like a sausage or snake, smooth and soft”, Type 5 being “Soft blobs with clear-cut edges, passes easily”, Type 6 being “Fluffy pieces with ragged edges, a mushy stool”, and Type 7 being “Watery, no solid pieces, entirely liquid”. The Gastrointestinal Symptoms Rating Scale (GSRS) [30], a 15-item clinical rating scale was completed a total of four times to assess symptoms related to gastrointestinal distress during the past week. Each item was scored on a seven-point scale where 1 = No Discomfort at all; 2 = Minor Discomfort; 3 = Mild Discomfort; 4 = Moderate Discomfort; 5 = Moderately Severe Discomfort; 6 = Severe Discomfort; 7 = Very Severe Discomfort. The GSRS encompasses areas such as: pain and discomfort in upper abdomen, heartburn, acid reflux, hunger pangs, nausea, stomach rumbling, bloating, burping, passing gas or flatus, constipation, diarrhea, hard stools, urgent need to have a bowel movement, and sensation of not completely emptying the bowels.

### 2.7. Stool Collection

Stool specimens were collected at four time points throughout the entire study protocol. The first two samples were collected before and after the first supplementation period while the final two samples were collected before and after the second supplementation period. All stool specimens were collected no more than 24-h prior to that study participant being scheduled to complete their laboratory visit. All samples were immediately frozen after sample collection by the study participants. Participants were instructed to keep their samples in the freezer and to remove them at the latest possible time to minimize thawing during transfer to the laboratory for storage. Participants were provided with all materials necessary to collect, store, and transport the stool specimen including commode fecal collection system, fecal collection tubes with screw top lids and scoops attached, disposable gloves, freezer packs, specimen labels, and transfer containers. Participants were instructed to raise the toilet seat and place the stool collection frame on the back of the toilet bowl and then lower the toilet seat down and place the collection bowl in frame. Participants then sat on the toilet and collected a stool sample in the container. Participants were instructed, if possible, not to urinate into the collection container. Participants then extracted a representative stool sample and filled each of the two provided sample collection tubes. Tubes were then secured, labeled, and frozen. All remaining stool was discarded into the toilet and flushed. Upon arrival in the laboratory each sample was immediately stored at −80 °C.

### 2.8. Fecal Microbiota Analysis

DNA was extracted from fecal samples by mechanical lysis with a FastPrep-24 (MP Biomedicals, Burlingame, CA, USA) and later purified using the DNA Fast Stool Minikit (Qiagen Inc. USA, Germantown, MD, USA) according to previously described methods [31]. The V4 region of bacterial 16S rRNA genes (294 bp) was amplified in 35 cycles according to previously described methods [32] with Takara ExTaq (Takara Bio USA, Mountain View, CA, USA) and the F515 (5′-GTGCCAGCMGCCGCGGTAA-3′) and R806 (5′-GGATACHVGGGTWTCTAAT-3′) primer containing random eight base pair barcode sequences on the 5′ end of the forward primer [33]. The Qubit Fluorometer (Thermo Fisher Scientific, San Francisco, CA, USA) was used to quantify PCR product concentrations prior to combining the PCR products in equal quantities for purification with the Wizard SV Gel and PCR Cleanup kit (Promega, Madison, WI, USA). The IonTorrent Fragment Plus Library Kit (Thermo Fisher) with AMPure XP Magnetic Beads (Beckman Coulter Life Sciences, Pasadena, CA, USA) was then used for DNA sequencing library preparation. Library purity and concentration was measured on a 2100 Bioanalyzer System (Agilent Technologies, Santa Clara, CA, USA). The library was diluted to 20 pM and then sequenced with an Ion Genestudio S5 System (Thermo Fisher).

Ion Torrent BAM files of raw DNA sequencing data were converted to the FASTQ format using BEDTools [34] and then analyzed using Quantitative Insights into Microbiology (QIIME2) version 2019.10 [35]. Reads shorter than 200 bp in length were removed. The sequences were demultiplexed using the cutadapt demux-single command [36], denoised using the QIIME deblur denoise-16S command [37], and truncated to a length of 270 bp to trim low q-score base positions. Multiple sequence Alignment using Fast Fourier Transform (MAFFT) was used for read alignment, while gapped and unconserved alignments were filtered using the alignment mask plugin [38]. A phylogenetic tree was generated using FastTree [39], and taxonomic classification was performed against the Greengenes 13.8 database [40]. Sequences of mitochondria and chloroplast origin were filtered from downstream analysis, leaving a total of 1,391,259 total high-quality reads associated with taxonomic annotations. Samples were rarified to 7874 reads for alpha and beta diversity analysis. Two samples were eliminated from the downstream analysis (GLC_3_L_Pre, GLC_7_L_Post), due to having low levels of annotated reads (GLC_7_L_Post: 8.3% reads annotated past kingdom level, GLC_3_L_Pre: 42.7% reads annotated past kingdom level). Beta diversity metrics of Bray-Curtis distances and Weighted and Unweighted Unifrac distances were plotted using principal coordinate analysis (PCoA) [32]. Alpha diversity metrics of Observed OTUs, Shannon Evenness, and Faith’s Phylogenetic Diversity were calculated.

### 2.9. Fecal Metabolome Analysis

Samples were manually homogenized with a sterile micro-spatula, and approximately 250 mg of fecal material was weighed and extracted using 1.5 mL of ice-cold Dulbecco’s phosphate buffered saline (DPBS, 1X, pH 7.4). After extraction, samples were centrifuged, and the pellet was dried using a Labconco FreeZone 4.5 L Freeze Dry System to determine fecal dry weight. The supernatant was sequentially filtered through a 0.22 m pore-size syringe filter (Millex-GP syringe filter, Millipore, Billerica, MA, USA) to remove microbes followed by a 3 kDa ultra-centrifugal filter (Amicon ultra-centrifugal filter, Millipore, Billerica, MA, USA) to remove any excess proteins. To 207 L of the filtrate, 23 L of an internal standard (5 mM DSS-d6 (trimethyl-silyl-propane-sulfonate) containing 0.2% NaN3 in 99.8% D2O was added to aid in quantification of metabolites. The pH of each sample was adjusted to 6.8 ± 0.1 through addition of small amounts of NaOH or HCl. Samples were transferred to 3 mm Bruker NMR tubes (Bruker, Brillerica, MA, USA) and stored at 4ºC until spectral acquisition. Nuclear magnetic resonance (NMR) spectra were acquired using a Bruker Avance 600 MHz NMR spectrometer equipped with a SampleJet autosampler (Bruker BioSpin, Billerica, MA USA) at 298 K using the NOESY 1H pre-saturation experiment (‘noesypr1d’). Data were acquired with a spectral width of 12 ppm, a 2.5-s acquisition time, a relaxation delay of 2.5 s, and a 100-millisecond mixing time using 32 transients and 8 dummy scans. Water saturation was applied during the relaxation delay and mixing time. Each spectrum was Fourier Transformed with zero filling to 128,000 data points, and the resulting Free Induction Decay (FID) was transformed with an exponential apodization function corresponding to a line-broadening of 0.5 Hz. Spectra were subsequently phased and baseline corrected using Chenomx NMRSuite Processor v8.3 (Chenomx Inc., Edmonton, AB, Canada). Metabolites were identified and quantified based on the known concentration of the internal standard, using Chenomx NMRSuite Profiler v8.3 based on the established method of targeted profiling [41]. All compounds in the database have been verified against known concentrations of reference NMR spectra of pure compounds, and this method has been shown to be reproducible and accurate [42,43]. The measured concentrations were corrected as described in He et al. [44].

### 2.10. Statistical Analysis

Primary outcomes for this study were considered to be between-group differences between the components of the GSRS and the Bristol Stool Chart with secondary outcomes being the metabolomic analysis. No between-gender analysis was completed due to low sample size. All analysis was completed with both genders collapsed using Microsoft Excel and the Statistical Package for the Social Sciences (v23; SPSS Inc., Chicago, IL, USA). Before any statistical tests were completed normality was assessed for all dependent variables. All non-normal data was log-transformed and then analyzed using both parametric and non-parametric approaches. For all dependent measures, descriptive statistics are presented herein as mean ± standard deviations. When the normality assumption was met, paired samples *t*-tests were computed using the post-pre changes for all variables and data was presented as means ± standard deviation. When the normality assumption was not met, Wilcoxon signed rank tests were completed using post-pre changes for all variables. For all statistical tests, data was considered statistically significant when the probability of type I error was 0.05 or less. A *p*-value between 0.05–0.10 was considered to be a statistical trend. Using this approach, no situations arose where the final statistical decision was different, whether a parametric or non-parametric approach was used. As such, between-group effect sizes, *p*-values, and 95% confidence intervals were computed to accompany traditional hypothesis testing outcomes.

To determine significant differences between bacterial alpha diversity metrics (the measure of microbiome diversity within a single sample), a paired *t*-test was used between alpha diversity values of different sample groups (*p* < 0.05). Significant differences in beta diversity (the measure of similarity or dissimilarity of multiple samples) between groups was also determined using the qiime diversity beta-group-significance command in QIIME2, which performs pairwise Permutational Multivariate Analysis of Variance (PERMANOVA) tests on beta diversity distance matrices (*p* < 0.05). Differential abundance of taxa between sample groups were evaluated using Multivariate Analysis by Linear Models (MaAsLin) in R version 4.0.2 [45] using the Maaslin2 command in the Maaslin2 R library [46] on rarified taxonomy data generated by QIIME2 (*p* < 0.05). Additional evaluation of differential abundance of taxa was performed using Analysis of Composition of Microbiomes (ANCOM) using the qiime composition ancom command after collapsing the feature table to the family level (level 5) using the qiime taxa collapse command on QIIME2 [47]. Data visualization was performed using the ggplot2 package, with data frame manipulation commands from the dplyr package, within the tidyverse collection of R packages [48].

## 3. Results

### 3.1. Adverse Events

No formalized approach to recording adverse events was completed primarily because gastrointestinal symptoms were being recorded four times throughout the study protocol. Nonetheless, participants were asked during each visit if they experienced any other adverse events associated with the research study that they needed to report and no other adverse events were reported.

### 3.2. Supplement Compliance

Compliance for the collection of dietary, physical activity data, and fecal samples were recorded throughout the protocol. Overall compliance for the entire study cohort across both conditions was calculated to be 86% with compliance exhibited by individual participants ranging from 50–100%. One male and one female participant during the glucosamine condition failed to turn in any diet, physical, or fecal sample compliance information. One female during the glucosamine condition, did not turn in food or physical activity records and in the placebo condition did not turn in any post-supplementation diet or physical activity records.

### 3.3. Baseline Differences

Eleven people completed the entire study protocol. In support of the crossover approach and washout creating homogenous cohorts at the start of supplementation, no differences in baseline levels of all dependent variables were observed (*p* = 0.13–1.000). Only one value (GSRS rating for Hard Stools) tended to be different (*p* = 0.08). Additionally, mixed factorial ANOVA revealed no significant order x condition interactions for all dependent variables.

### 3.4. Participant Demographics and Hemodynamics

Table 1 contains body mass, sleep, physical activity, hemodynamic, and dietary data for all participants. No between-group or within-group differences were identified for any of these variables.

### 3.5. Bristol Stool Chart

No significant changes in Bristol Stool chart ratings were observed within the PLA (*p* = 0.10) and GLU (*p* = 0.10) or between group (*p* = 0.20) (Table 2).

### 3.6. Gastrointestinal Symptom Rating Scale

Significantly greater increases in stomach bloating (GSRS7) were reported in the PLA group when compared to changes observed in GLU (condition x time, *p* = 0.03). Additionally, stomach bloating ratings in PLA significantly increased (*p* = 0.03) from baseline while no changes were observed (*p* = 0.32) in GLU. Both ‘Constipation’ (GSRS10, *p* = 0.10) and ‘Hard Stools’ (GSRS13, *p* = 0.08) exhibited a trend for the changes observed during supplementation to be different between groups. Within-group changes for ‘Constipation’ indicated a decrease in GLU (Pre: 1.82 ± 1.4 vs. Post: 1.09 ± 0.3, *p* = 0.10) while values reported for PLA increased (Pre: 1.09 ± 0.3 vs. Post: 1.36 ± 0.5, *p* = 0.18). Additionally, within-group changes for ‘Hard Stools’ showed a larger decrease in GLU (Pre: 1.55 ± 1.04 vs. Post: 1.09 ± 0.3, *p* = 0.10) when compared to changes observed in PLA (Pre: 1.27 ± 0.65 vs. Post: 1.09 ± 0.3, *p* = 0.41). No other GSRS variables exhibited changes between groups as a result of supplementation. Notably, other GSRS variables for both PLA and GLU exhibited tendencies (*p* = 0.05 to 0.10) to change from their baseline scores. ‘Constipation Syndrome’ exhibited a trend for the changes observed between GLU and PLA to be different (*p* = 0.06). In this respect, changes in GLU tended to decrease from baseline (Pre: 5.09 ± 3.45 vs. Post: 3.27 ± 0.9, *p* = 0.07) while no change was observed in PLA (Pre: 3.73 ± 1.27 vs. Post: 3.64 ± 0.81, *p* = 0.93). Interestingly, both PLA and GLU observed similar increases (a negative change) in the ingestion syndrome (*p* = 0.07 for both GLU and PLA), but no between-group changes were observed (*p* = 0.88). All other GSRS changes are observed in Table 2. One male participant during the Glucosamine condition self-reported increased frequency and severity of diarrhea secondary to international travel during his supplementation protocol. His results have not been removed from the analysis.

### 3.7. Fecal Microbiota Diversity

**Figure 3:**
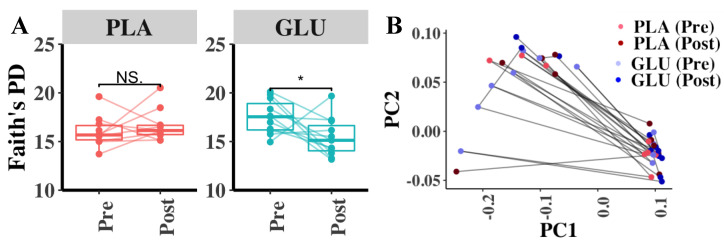
Intestinal microbiota diversity before and after glucosamine consumption. (**A**) Faith’s Phylogenetic Diversity of bacterial contents in fecal samples before and after PLA and GLU consumption, with points representing individual fecal sample communities. Bacterial composition before and after GLU treatment was significantly different (* Student *t*-test, *p* < 0.05). (**B**) Principal Coordinates Analysis (PCoA) of bacterial beta-diversity according to the unweighted UniFrac metric. The solid black lines connect the fecal microbiota from one individual at each of the four fecal collection time points. The dots for each condition are color coordinated whereby the light and dark blue dots are the Pre and Post-Supplementation GLU samples, respectively. Similarly, the pink and dark red dots are Pre and Post PLA samples, respectively.

Longitudinal analysis showed that the alpha diversity of the bacterial communities in the fecal contents was significantly decreased following GLU consumption compared to baseline values (Faith’s Phylogenetic Diversity (PD), paired *t*-test, treatment + time, *p* = 4.59 × 10^−3^) (Figure 3A). By comparison, Faith’s PD did not change over time when PLA was consumed (Figure 3A). The other alpha diversity measures (Observed OTUs or Shannon Evenness) were not significantly altered by the inclusion of either GLU or PLA into the diet (*p >* 0.05, paired *t*-test). Although bacterial beta diversity as assessed by unweighted Unifrac distances showed that the fecal microbiota changed over time between fecal samples collected before and after GLU or PLA consumption (Figure 3B), the changes in beta-diversity were not significantly different compared to baseline (PERMANOVA, *p* > 0.05). No significant differences in fecal microbiota diversity according to the weighted Unifrac and Bray-Curtis Dissimilarity metrics (*p* > 0.05, PERMANOVA) were found.

**Figure 4:**
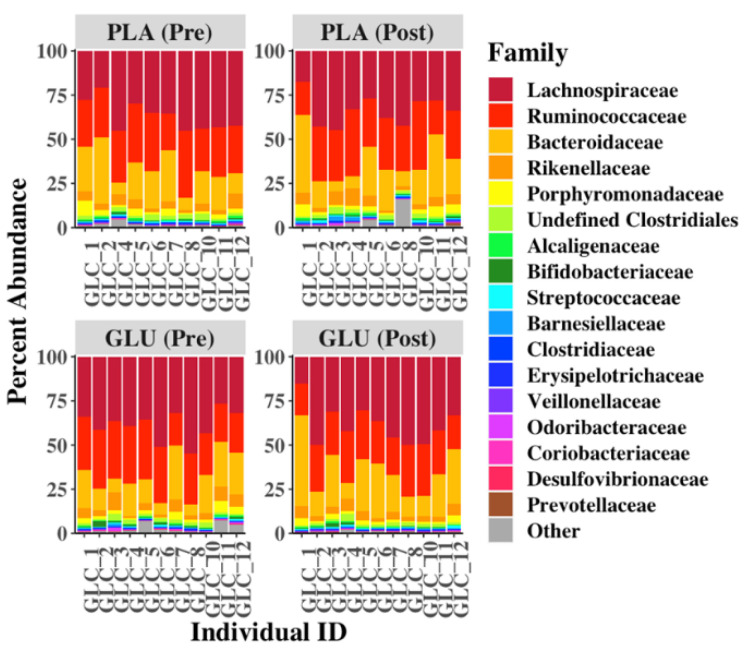
Relative abundance of bacteria shown at the family level. Proportions of individual bacterial families pre- and post-GLU and -PLA consumption. ASVs were combined if their family-level taxonomic designations were identical. The seventeen most abundant bacterial families are annotated, with all other taxa grouped under the annotation of “Other”.

**Figure 5:**
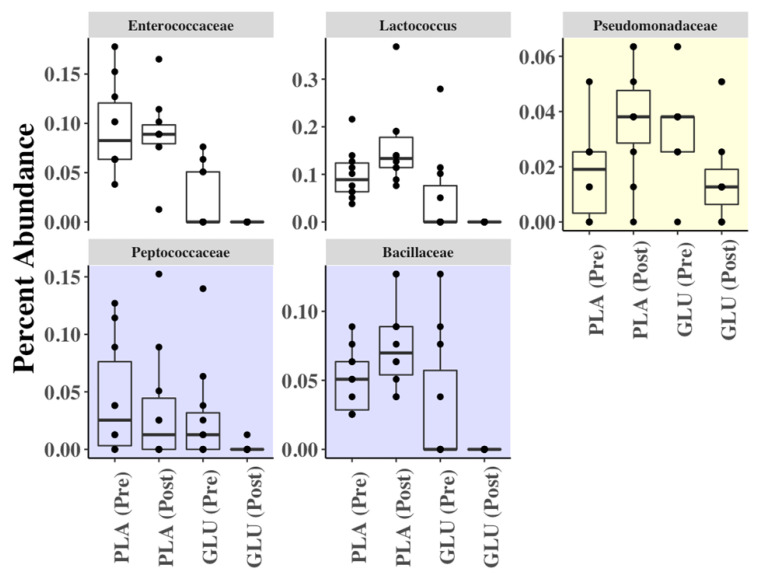
Bacterial taxa present in significantly different proportions in fecal contents before and after PLA and GLU supplementation. The white background for *Enterococcaceae* and *Lactococcus* indicates that these taxa were present in significantly different proportions between PLA and GLU (PRE). The yellow background for *Pseudomonadaceae* denotes a significant difference between GLU (Pre) and GLU (Post). The purple background for *Peptococcaceae* and *Bacillaceae* denotes a significant difference for GLU (Post) compared to GLU (Pre) as well as to both PLA (Pre) and PLA (Post). Taxa were compared using MaAsLin (*p* < 0.05).

*Lachnospiraceae* and *Ruminococcaceae* (Firmicutes) and *Bactroidaceae* (Bacteroidetes) families comprised the majority of bacterial taxa present in the fecal contents (84.8 ± 4.7% combined relative abundance) (Figure 4). No other bacterial families were present at an average relative abundance higher than 5% in any of the fecal samples. Comparisons between the fecal microbiota contents showed that the levels of *Enterococcaceae* and *Lactococcus* were variable, but not in a manner that was associated with GLU consumption (Figure 5). Among the other taxa identified, lower proportions of *Pseudomonadaceae* (*p* = 0.014, Log_2_ FC = −1.05), *Peptococcaceae* (*p* = 0.021, Log_2_ FC = −4.52), and *Bacillaceae* (*p* = 0.038, not detected post-GLU), and were found after GLU intake compared to baseline (GLU (Pre)) (Figure 5). By comparison, there were no significant differences in bacterial composition after PLA ingestion compared to PLA (Pre) (MaAsLin *p* > 0.05). Additionally, levels of *Peptococcaceae* and *Bacillaceae* were also significantly reduced after GLU intake compared to either before (PLA (Pre)) or after PLA (PLA (Post)) consumption (Figure 5). While the proportions of those two families were also lower at GLU (Pre) baseline compared to the PLA (Post) time point, those differences were not significant (MaAsLin, *p* > 0.05).

### 3.8. Fecal Metabolome

GLU and PLA supplementation had no effect on individual or total short-chain fatty acids (*p* > 0.05) (Table 3).

Compared to baseline, GLU supplementation significantly reduced fecal glutamate, isoleucine, leucine and valine content (*p* < 0.05), as well as total branched-chain amino acid (−24%, *p* < 0.05), and total amino acids (−21%, *p* < 0.05). No significant changes were observed for any of the individual or total amino acids in the control group (*p* > 0.05) (Table 4).

PLA supplementation did not result in any significant changes of miscellaneous fecal metabolites (*p* > 0.05) (Table 5). GLU supplementation significantly reduced nucleotides compared to baseline (uracil, *p* = 0.02), and between groups (ribose, *p* = 0.01 and uracil, *p* = 0.04). No GLU was detected in either group.

## 4. Discussion

This study sought to determine the impact of a three-week glucosamine supplementation protocol on changes in stool quality, gastrointestinal symptoms, and microbiome diversity, enrichment, and function. Our primary findings indicate that glucosamine administration resulted in favorable changes in stomach bloating in comparison to placebo, while no changes in stool quality were observed. Additionally, changes in constipation and hard stools tended to exhibit between-group differences that favored glucosamine supplementation. Modest but significant alterations in bacterial composition in the fecal samples were observed with glucosamine consumption, including reductions in alpha diversity and reduced proportions of several bacterial taxa. These changes occurred in the absence of observable modifications to the fecal metabolome. The findings of this study suggest that benefits of glucosamine consumption may be partially related to modification of the intestinal environment including the gut microbiota. Results from the present investigation are unique as they represent the first placebo-controlled data involving just glucosamine supplementation in healthy subjects. In this respect, previous data by Coulson et al. [25] who had osteoarthritic patients supplement for 12 weeks with 3000 mg of glucosamine align with our outcomes demonstrating some improvements in markers of gastrointestinal symptoms.

The reduction in bacterial alpha-diversity (a metric of the total bacterial contents in the fecal samples) with the inclusion of glucosamine in the diet is consistent with previous reports where both glucosamine and chondroitin [26] or glucosamine and green lipped muscle extract [25] were consumed. However, our other findings differed from these studies including a lack of an effect on bacterial beta-diversity and differences between the affected taxa compared with those prior reports. Specifically, we found significant reductions in the proportions of *Pseudomonadaceae*, *Peptococcaceae*, and *Bacillaceae* in the fecal contents when glucosamine was consumed compared to baseline samples. It is also notable that the levels of *Peptococcaceae* and *Bacillaceae* were also significantly reduced after GLU compared to before and after PLA consumption, thereby indicating a longitudinal and treatment specific effect. Conversely, despite reductions in the proportions of these taxa, there was no single bacterial genus or family that was enriched in the present study. These findings may be due to glucosamine-mediated inhibition of certain bacterial taxa and the concurrent (non-specific) enrichment of multiple bacterial species able to metabolize this compound. Lastly, the cross-over, longitudinal design study and inclusion of baseline samples showed how the bacterial contents within a single individual can change over time. Comparisons of the baseline samples indicated significant variations in the abundance of *Enterococcaceae* and *Lactococcus* suggesting that other factors may determine the levels of these taxa in the distal intestine. While Navarro et al. [26] recruited healthy subjects, the differences in the supplementation protocol (14 vs. 21 days, combination of glucosamine + chondroitin vs. glucosamine alone, 1500 mg vs. 3000 mg dose and frequency of glucosamine, and the source of glucosamine (shellfish-derived vs. fermentation)) may have led to differences in our observed outcomes.

No GLU was detected in either group in the fecal metabolome analysis. The origin of N-acetylglucosamine in the feces is not clear and is likely not to be derived from the supplementary glucosamine, as N-acetylglucosamine is made by bacteria and used for cell wall synthesis, or by the host, and is part of the extracellular matrix. Therefore, the origin of this compound in the feces is not clear. Lower BCAA, total amino acids, and glutamate could indicate increased absorption of those nutrients, increased utilization by microbes, or decreased production, assuming that some of the amino acids come directly from gut bacteria. Additionally, reduced nitrogen excretion could indicate reduced muscle protein breakdown, potentially due to reduced inflammation. Gut microbiota-derived metabolites affect many biological processes of the host, including appetite control and weight management. Amino acids are among the gut microbiota-derived metabolites which have previously demonstrated alterations in obesity, indicating potential alternative health applications for glucosamine [49].

Future research is needed to better identify an optimal dosing regimen and to see if other populations exhibit similar or different responses to our measured outcomes. Strengths from our investigation center upon it being the first randomized, double-blind, placebo-controlled trial to examine the impact of acute glucosamine supplementation on changes in fecal microbiota and the metabolome, in conjunction with changes in stool quality and gastrointestinal outcomes. Our crossover design further limited between-group variability and our dietary control approach leading up to the collection of each sample was consistent with other study designs of this nature. However, our study was not free of limitations as longer supplementation periods and greater sample sizes should be investigated in future studies.

## 5. Conclusions

In conclusion, results from the first study to examine the impact of three weeks of non-shellfish derived glucosamine supplementation against a placebo in a randomized, crossover fashion in healthy men and women revealed significant improvements in functional outcomes such as stomach bloating in addition to a favorable trend towards improvements in ratings of constipation and hard stools. Bacterial diversity in the fecal contents was significantly decreased following GLU consumption compared to baseline values. GLU supplementation significantly reduced individual and total branched-chain amino acid excretion, with no effect on fecal short-chain fatty acid levels.

## Figures and Tables

**Table 1:** Participant Demographics, Hemodynamics, and Dietary Intake.

	PLA			GLU			PLA vs. GLU
	Pre	Post	*p*	Pre	Post	*p*	*p*
Body Mass (kg)	77.0 ± 13.3	76.6 ± 12.6	0.15	76.5 ± 12.7	76.8 ± 12.5	0.60	0.43
Heart Rate (beats/min)	66.8 ± 10.4	65.8 ± 7.9	0.69	66.8 ± 7.7	63.1 ± 8.1	0.22	0.62
Systolic BP (mmHg)	123.6 ± 12.2	116.7 ± 13.3	0.04	121.7 ± 14.2	119.4 ± 10.8	0.60	0.09
Diastolic BP (mmHg)	74.3 ± 7.0	72.1 ± 8.2	0.02	73.9 ± 9.3	77.0 ± 9.8	0.33	0.11
Physical Activity (min/day)	51 ± 29	53 ± 29	0.90	45 ± 24	32 ± 20	0.33	0.56
Energy (kcal/day)	2037 ± 621	2091 ± 742	0.32	2146 ± 663	2085 ± 530	0.82	0.92
Carbohydrate (g/day)	213 ± 50	210 ± 91	0.76	209 ± 69	189 ± 36	0.80	0.82
Protein (g/day)	124 ± 68	118 ± 51	0.78	124 ± 52	123 ± 46	0.93	0.90
Fat (g/day)	75 ± 28	87 ± 35	0.08	89 ± 14	91 ± 36	0.35	0.86

Placebo (PLA); Glucosamine (GLU).

**Table 2:** Bristol Stool Chart and Gastrointestinal Symptom Rating Scale.

	PLA				GLU				PLA vs. GLU			
	Pre	Post	*p*	*d*	Pre	Post	*p*	*d*	*p* *	%	*d*	95% CI
Bristol Stool	3.1 ± 0.8	4.0 ± 1.4	0.10	0.79	3.3 ± 0.8	3.6 ± 0.8	0.10	0.38	0.20	−19.9	−0.61	(−1.83, 0.74)
GSRS1	1.3 ± 0.6	1.5 ± 0.7	0.08	0.31	1.2 ± 0.4	1.5 ± 0.7	0.18	0.53	1.00	31.3	0.69	(−0.42, 0.42)
GSRS2	1.0 ± 0.0	1.1 ± 0.3	0.32	0.47	1.1 ± 0.3	1.2 ± 0.4	0.56	0.28	1.00	−0.9	0.00	(−0.30, 0.30)
GSRS3	1.0 ± 0.0	1.0 ± 0.0	1.00	0.00	1.0 ± 0.0	1.2 ± 0.4	0.16	0.71	0.16	20.0	1.00	(−0.09, 0.45)
GSRS4	1.6 ± 0.7	1.2 ± 0.4	0.05	0.70	1.8 ± 1.0	1.2 ± 0.4	0.08	0.79	0.45	−11.3	−0.41	(−1.07, 0.53)
GSRS5	1.5 ± 1.2	1.3 ± 0.5	0.71	0.22	1.3 ± 0.9	1.6 ± 1.2	0.32	0.28	0.33	35.4	0.48	(−0.46, 1.37)
GSRS6	1.1 ± 0.3	1.2 ± 0.4	0.32	0.28	1.5 ± 0.7	2.2 ± 1.3	0.10	0.67	0.16	37.6	0.77	(−0.32, 1.41)
GSRS7	1.7 ± 1.3	2.4 ± 1.3	0.03	0.54	1.9 ± 1.2	2.1 ± 1.2	0.32	0.17	0.03	−30.7	−0.40	(−0.81, −0.10)
GSRS8	1.0 ± 0.0	1.1 ± 0.3	0.32	0.47	1.2 ± 0.4	1.2 ± 0.4	1.0	0.00	0.56	−10	−0.31	(−0.45, 0.27)
GSRS9	2.1 ± 1.1	2.6 ± 1.5	0.25	0.38	1.8 ± 0.8	2.5 ± 1.2	0.08	0.69	0.87	8.8	0.08	(−1.51, 1.69)
GSRS10	1.1 ± 0.3	1.4 ± 0.5	0.18	0.73	1.8 ± 1.4	1.1 ± 0.3	0.10	0.69	0.10	−64.9	−1.29	(−2.20, 0.20)
GSRS11	1.0 ± 0.0	1.2 ± 0.4	0.16	0.71	1.0 ± 0.0	1.7 ± 1.6	0.11	0.62	0.29	55.0	0.68	(−0.55, 1.64)
GSRS12	***	***	***	***	***	***	***	***	***	***	***	***
GSRS13	1.3 ± 0.7	1.1 ± 0.3	0.41	0.37	1.6 ± 1.0	1.1 ± 0.3	0.10	0.68	0.08	−15.5	−0.43	(−0.59, 0.04)
GSRS14	1.0 ± 0.0	1.1 ± 0.3	0.32	0.47	1.3 ± 0.9	1.6 ± 0.9	0.08	0.33	0.16	13.0	0.29	(−0.09, 0.45)
GSRS15	1.4 ± 0.7	1.2 ± 0.4	0.48	0.35	1.7 ± 1.1	1.1 ± 0.3	0.07	0.74	0.20	−23.8	−0.67	(−1.21, 0.30)
Diarrhea	2.0 ± 0.0	2.3 ± 0.7	0.18	0.61	2.3 ± 0.9	3.3 ± 1.6	0.02	0.77	0.13	30.6	0.76	(−0.36, 1.81)
Ingestion	5.9 ± 2.1	7.3 ± 2.8	0.09	0.57	6.5 ± 2.5	7.9 ± 3.0	0.07	0.51	0.88	−0.4	0.04	(−2.52, 2.70)
Constipation	3.7 ± 1.3	3.6 ± 0.8	0.93	0.09	5.1 ± 3.5	3.3 ± 0.9	0.07	0.70	0.06	−33.3	−0.89	(−3.70, 0.24)
Abdom Pain	4.3 ± 1.9	4.0 ± 1.2	0.55	0.19	4.3 ± 1.7	4.2 ± 1.7	0.67	0.06	0.86	4.2	0.11	(−1.05, 1.41)
Reflux	2.0 ± 0.0	2.1 ± 0.3	0.32	0.47	2.1 ± 0.3	2.4 ± 0.7	0.18	0.56	0.16	8.4	0.45	(−0.09, 0.45)

Values inside table are means ± standard deviations for each specified variable and timepoint. Bold-type face = Between-group difference (*p* < 0.05). * = *p*-value computed using non-parametric statistical approaches (Wilcoxon-signed rank test). GSRS1 = Pain and discomfort in upper abdomen or stomach; GSRS2 = Heartburn; GSRS3 = Acid reflux; GSRS4 = Hunger pangs; GSRS5 = Nausea; GSRS6 = Stomach rumbling; GSRS7 = Stomach bloating; GSRS8 = Burping; GSRS9 = Passing gas or flatulence; GSRS10 = Constipation; GSRS11 = Diarrhea; GSRS 12 = (***) Was not assessed due to technical error; GSRS13 = Hard stools; GSRS14 = Urgent need to have a bowel movement; GSRS15 = Sensation of not completely emptying the bowels; Diarrhea = Combined score of GSRS11 and GSRS14; Ingestion = Combined score of GSRS6, GSRS7, GSRS8, and GSRS9; Constipation = Combined score of GSRS10, GSRS13, and GSRS14; Abdom Pain = Combined score of GSRS 1, GSRS4, and GSRS5; Reflux = Combined score of GSRS2 and GSRS3.

**Table 3:** Fecal Short-Chain Fatty Acids.

						PLA vs. GLU			
		PLA		GLU		*p* ^‡^	%	*d*	95% CI
Formate	Pre	244 ± 116	*d* = 0.09	298 ± 166	*d* = 0.43	0.25	30.7	0.49	(−270, 81)
	Post	255 ± 135	*p* * = 0.56	403 ± 299	*p* * = 0.20				
Acetate	Pre	55,604 ± 17,431	*d* = 0.19	40,675 ± 12,876	*d* = 0.25	0.90	0.5	−0.05	(−14,026, 15,644)
	Post	59,417 ± 22,185	*p* * = 0.70	43,680 ± 11,351	*p* * = 0.46				
Propionate	Pre	15,419 ± 8193	*d* = 0.18	12,596 ± 4145	*d* = 0.01	0.48	−10.0	−0.22	(−3303, 6411)
	Post	17,040 ± 9419	*p* * = 0.86	12,663 ± 5766	*p* * = 0.96				
Butyrate	Pre	15,244 ± 9517	*d* = 0.08	11,370 ± 5781	*d* = 0.35	0.51	14.3	0.19	(−6234, 3366)
	Post	15,978 ± 7809	*p* * = 0.87	13,538 ± 6558	*p* * = 0.14				
Iso-butyrate	Pre	1787 ± 984	*d* = 0.45	2097 ± 887	*d* = 0.12	0.11	−38.6	−0.59	(−193, 1612)
	Post	2356 ± 1474	*p* * = 0.52	1956 ± 1334	*p* * = 0.73				
Isovalerate	Pre	1542 ± 967	*d* = 0.37	1885 ± 886	*d* = 0.16	0.09	−18.9	−0.33	(−365, 1377)
	Post	1956 ± 1226	*p* * = 0.68	1712 ± 1304	*p* * = 0.67				
Valerate	Pre	2674 ± 1362	*d* = 0.24	2691 ± 1314	*d* = 0.08	0.22	−36.0	−0.53	(−110, 1283)
	Post	3063 ± 1833	*p* * = 0.80	2574 ± 1519	*p* * = 0.78				
Total SCFA	Pre	92,514 ± 35,299	*d* = 0.20	71,612 ± 22,891	*d* = 0.20	0.81	−1.3	−0.08	(−21,884, 27,156)
	Post	100,064 ± 41,479	*p* * = 0.80	76,527 ± 25,492	*p* * = 0.49				

Values inside table are means ± standard deviations for each specified variable and time point. * = *p*-value computed using paired samples *t*-test. ^‡^ *p*-value computed using 2 × 2 within-within factorial ANOVA.

**Table 4:** Fecal Amino Acids.

						PLA vs. GLU			
		PLA		GLU		*p* ^‡^	*%*	*d*	95% CI
Alanine	Pre	2503 ± 1098	*d* = 0.31	2667 ± 1134	*d* = 0.46	0.24	−33.4	−0.72	(−688, 2415)
	Post	2928 ± 1636	*p* * = 0.62	2228 ± 721	*p* * = 0.16				
Glutamate	Pre	7857 ± 2856	*d* = 0.20	8882 ± 3831	*d* = 0.78	0.01	−30.7	−0.90	(801, 4504)
	Post	8400 ± 2641	*p* * = 0.68	6773 ± 2223	*p* * = 0.04				
Glycine	Pre	1082 ± 438	*d* = 0.27	1207 ± 514	*d* = 0.53	0.22	−34.5	−0.76	(−293, 1091)
	Post	1238 ± 684	*p* * = 0.63	965 ± 399	*p* * = 0.09				
Isoleucine	Pre	904 ± 402	*d* = 0.28	1029 ± 462	*d* = 0.59	0.17	−40.5	−0.84	(−202, 995)
	Post	1049 ± 603	*p* * = 0.50	777 ± 388	*p* * = 0.05				
Leucine	Pre	1303 ± 611	*d* = 0.10	1422 ± 622	*d* = 0.58	0.23	−28.4	−0.64	(−307, 1101)
	Post	1372 ± 737	*p* * = 0.68	1094 ± 496	*p* * = 0.05				
Lysine	Pre	1658 ± 748	*d* = 0.22	1834 ± 815	*d* = 0.55	0.07	−32.1	−0.73	(−56, 1194)
	Post	1847 ± 943	*p* * = 0.59	1454 ± 554	*p* * = 0.11				
Methionine	Pre	374 ± 367	*d* = 0.08	424 ± 290	*d* = 0.28	0.55	−23.7	−0.18	(−263, 458)
	Post	399 ± 292	*p* * = 0.84	352 ± 210	*p* * = 0.15				
Phenylalanine	Pre	625 ± 311	*d* = 0.04	629 ± 280	*d* = 0.34	0.52	−15.6	−0.34	(−237, 434)
	Post	638 ± 331	*p* * = 0.70	544 ± 208	*p* * = 0.26				
Proline	Pre	709 ± 304	*d* = 0.02	687 ± 280	*d* = 0.11	0.85	−5.7	−0.12	(−413, 493)
	Post	716 ± 383	*p* * = 0.99	654 ± 326	*p* * = 0.63				
Threonine	Pre	768 ± 311	*d* = 0.22	831 ± 355	*d* = 0.56	0.16	−31.8	−0.73	(−127, 641)
	Post	853 ± 455	*p* * = 0.53	659 ± 252	*p* * = 0.14				
Tyrosine	Pre	789 ± 468	*d* = 0.21	874 ± 394	*d* = 0.49	0.16	−30.5	−0.66	(−128, 640)
	Post	879 ± 387	*p* * = 0.60	707 ± 273	*p* * = 0.14				
Valine	Pre	1180 ± 507	*d* = 0.30	1351 ± 610	*d* = 0.65	0.15	−42.4	−0.90	(−239, 1329)
	Post	1376 ± 791	*p* * = 0.46	1003 ± 451	*p* * = 0.03				
Total BCAAs	Pre	3386 ± 1502	*d* = 0.22	3802 ± 1674	*d* = 0.62	0.18	−36.5	−0.80	(−3413, 735)
	Post	3796 ± 2123	*p* * = 0.54	2873 ± 1312	*p* * = 0.04				
Total AA	Pre	19,752 ± 7608	*d* = 0.24	21,838 ± 8104	*d* = 0.68	0.06	−31.0	−0.89	(−246, 13,390)
	Post	21,694 ± 8345	*p* * = 0.59	17,208 ± 5190	*p* * = 0.02				

Values inside table are means ± standard deviations for each specified variable and time point. Bold-type face = Between-group difference (*p* < 0.05). * = *p*-value computed using paired samples *t*-test. ^‡^ *p*-value computed using 2 × 2 within-within factorial ANOVA.

**Table 5:** Misc. Fecal Metabolites.

						PLA vs. GLU			
		PLA	*p* *	GLU	*p* *	*p* ^‡^	%	*d*	95% CI
Amino Acid Breakdown Products									
Cadaverine	Pre	157 ± 76	*d* = 0.58	170 ± 52	*d* = 0.09	0.23	−29.1	−0.73	(−35, 128)
	Post	198 ± 66	*p* * = 0.30	165 ± 56	*p* * = 0.80				
Putrescine	Pre	90 ± 84	*d* = 0.17	92 ± 59	*d* = 0.38	0.88	5.0	0.06	(−79, 69)
	Post	108 ± 120	*p* * = 0.69	115 ± 61	*p* * = 0.20				
p-Cresol	Pre	464 ± 298	*d* = 0.25	597 ± 330	*d* = 0.10	0.29	−27.7	−0.34	(−144, 421)
	Post	557 ± 421	*p* * = 0.98	551 ± 537	*p* * = 0.72				
Urocanate	Pre	103 ± 29	*d* = 0.46	104 ± 57	*d* = 0.36	0.17	−38.7	−0.81	(−21, 100)
	Post	124 ± 58	*p* * = 0.21	85 ± 48	*p* * = 0.30				
Nucleotide Breakdown Products									
Hypoxanthine	Pre	392 ± 224	*d* = 0.13	414 ± 190	*d* = 0.53	0.22	−27.1	−0.59	(−83, 317)
	Post	420 ± 222	*p* * = 0.73	325 ± 143	*p* * = 0.06				
Ribose	Pre	1110 ± 552	*d* = 0.74	1428 ± 760	*d* = 0.15	0.01	−37.4	−0.77	(158, 714)
	Post	1450 ± 339	*p* * = 0.07	1332 ± 533	*p* * = 0.65				
Uracil	Pre	866 ± 425	*d* = 0.17	922 ± 352	*d* = 0.74	0.04	−34.7	−0.81	(26, 604)
	Post	943 ± 469	*p* * = 0.65	684 ± 287	*p* * = 0.02				
Microbially Produced Fermentation Products									
Ethanol	Pre	1293 ± 2442	*d* = 0.45	169 ± 98	*d* = 0.69	0.33	123.7	0.71	(−2940, 1108)
	Post	481 ± 784	*p* * = 0.43	272 ± 187	*p* * = 0.04				
Fumarate	Pre	175 ± 75	*d* = 0.41	225 ± 151	*d* = 0.05	0.40	−29.4	−0.43	(−84, 190)
	Post	221 ± 139	*p* * = 0.19	218 ± 115	*p* * = 0.84				
Lactate	Pre	38 ± 20	*d* = 0.50	60 ± 49	*d* = 0.40	0.56	107.7	0.40	(−394, 229)
	Post	68 ± 82	*p* * = 0.47	172 ± 396	*p* * = 0.43				
Methionine-sulfoxide	Pre	241 ± 166	*d* = 0.19	258 ± 191	*d* = 0.33	0.29	37.4	−0.51	(−95, 282)
	Post	278 ± 222	*p* * = 0.42	202 ± 149	*p* * = 0.39				
Phenylacetate	Pre	888 ± 577	*d* = 0.28	1095 ± 511	*d* = 0.27	0.22	−30.6	−0.4	(−210, 791)
	Post	1082 ± 772	*p* * = 0.78	999 ± 786	*p* * = 0.70				
Succinate	Pre	924 ± 642	*d* = 0.15	635 ± 289	*d* = 0.34	0.94	8.0	0.03	(−497, 462)
	Post	1029 ± 712	*p* * = 0.99	758 ± 430	*p* * = 0.48				
Likely Host Derived									
3-Hydroxybutyrate	Pre	74 ± 37	*d* = 0.35	89 ± 62	*d* = 0.26	0.98	4.3	0.02	(−60, 58)
	Post	60 ± 42	*p* * = 0.53	76 ± 35	*p* * = 0.41				
N-Acetylglucosamine	Pre	260 ± 216	*d* = 0.12	249 ± 147	*d* = 0.41	0.56	−34.9	−0.48	(−248, 425)
	Post	286 ± 207	*p* * = 0.81	187 ± 152	*p* * = 0.16				
Likely Diet Derived									
Glucosamine	Pre	n.d.		n.d.					
	Post	n.d.		n.d.					
Glucose	Pre	3650 ± 3069	*d* = 0.16	2229 ± 2054	*d* = 0.45	0.38	61.4	0.59	(−5329, 2252)
	Post	3217 ± 2414	*p* * = 0.90	3334 ± 2753	*p* * = 0.23				
Glycerol	Pre	6013 ± 2267	*d* = 0.22	7458 ± 7347	*d* = 0.18	0.55	−33.0	−0.40	(−5965, 10,342)
	Post	7141 ± 7055	*p* * = 0.62	6397 ± 3575	*p* * = 0.47				
Nicotinate	Pre	371 ± 153	*d* = 0.31	327 ± 162	*d* = 0.41	0.76	−6.6	−0.13	(−105, 139)
	Post	332 ± 95	*p* * = 0.27	271 ± 106	*p* * = 0.23				
Xylose	Pre	594 ± 317	*d* = 0.23	441 ± 420	*d* = 0.54	0.23	70.8	0.82	(−912, 250)
	Post	525 ± 271	*p* * = 0.83	702 ± 545	*p* * = 0.31				

Values inside table are means ± standard deviations for each specified variable and time point. Bold-type face = Between-group difference (*p* < 0.05). * = *p*-value computed using paired samples *t*-test. ^‡^ *p*-value computed using 2 × 2 within-within factorial ANOVA. n.d. = non detectable.

## Data Availability

The datasets used and/or analyzed during the current study are available from the corresponding author upon reasonable request. Access to microbiota data has been made publicly available at European Bioinformatics Institute (EMBL-EBI) with accession number ERP128748.
